# CSF LPV concentrations and viral load in viral suppressed patients on LPV/r monotherapy given once daily

**DOI:** 10.7448/IAS.17.4.19587

**Published:** 2014-11-02

**Authors:** Juan Tiraboschi, Arkaitz Imaz, Elena Ferrer, Maria Saumoy, Nerea Rozas, Marga Maso, Antonia Vila, Jordi Niubo, Daniel Podzamczer

**Affiliations:** 1 HIV Unit, Infectious Disease, Hospital Universitari de Bellvitge, Barcelona, Spain; 2 HIV Unit, Hospital Universitari de Bellvitge, Barcelona, Spain; 3 Microbiology Service, Hospital Universitari de Bellvitge, Barcelona, Spain

## Abstract

**Introduction:**

Plasma trough concentrations of lopinavir (LPV) given as LPV/r 800/200 mg once daily (OD) are reduced in comparison with 400/100 mg twice daily (BID). While OD dosage of LPV/r is sufficient to achieve viral suppression in plasma, data about drug penetration and viral suppression in central nervous system (CNS) is needed, mainly if LPVr is used as maintenance monotherapy strategy in selected patients. The objective of this study was to evaluate CSF HIV-1 RNA and CSF LPV concentrations in patients receiving LPV/r monotherapy OD (LPVrMOD).

**Material and Methods:**

This is a cross-sectional sub-study within a prospective, open-label pilot simplification study to evaluate the efficacy and safety of LPV/rMOD in virologically suppressed patients previously receiving a BID LPV/r monotherapy regimen (LPV/rMBID), the “Kmon study” (NCT01581853). To assess LPV concentrations and HIV-1 RNA in CSF, a lumbar puncture (LP) was performed in a subgroup of patients after at least one month of LPVrMOD treatment. Plasma-paired samples of all patients were also obtained. HIV-1 RNA was determined by real-time PCR (limit of detection 40 copies/mL). Liquid chromatography-tandem mass spectrometry (Tandem labs, NJ) was used to determine CSF and blood plasma LPV concentrations.

**Results:**

Nine patients were included. Median (range) age was 48 (34–56) years, median CD4 cell count 672 (252–1,408) cells/mL, median nadir CD4 count 125 (35–537) cells/mL and 40% of subjects were HCV-positive. Before starting LPV/rMOD median time on a LPV/r-containing regimen and on LPV/rMBID were 9 (4–11) years and 15 (7–24) months respectively, median time with undetectable HIV viral load was 5 (3–12) years and 2 patients had a previous documented blip. LP was performed a median of 24 (8–36) weeks after starting LPV/rMOD and 24 (11–28) hours after the last LPV/rMOD dose CSF and plasma HIV RNA was 40 copies/mL in all patients. Median LPV CSF concentration was 9.78 (1.93–78.3) ng/mL, median LPV plasma concentration 1,103 (377–16,700) ng/mL and median LPV CSF/plasma ratio 0.3% (0.1–1.2).

**Conclusions:**

No CSF viral escape was detected and LPV concentrations were above the IC50 for wtHIV-1 (1.9 ng/mL). However, as concentrations were close to IC50 in some patients, a careful clinical follow up of patients receiving this regimen would be advisable. Larger longitudinal studies will be helpful for a better understanding of the CNS antiviral activity of LPVr monotherapy.

